# Supplementary material for: Synergistic cooperation promotes multicellular performance and unicellular free-rider persistence
Source: Nat Commun. 2017 Jun 5;8:15707. doi: 10.1038/ncomms15707 (PMC5465372; doi:10.1038/ncomms15707)
Supplement: Supplementary Information — Supplementary Figures [file ncomms15707-s1.pdf]

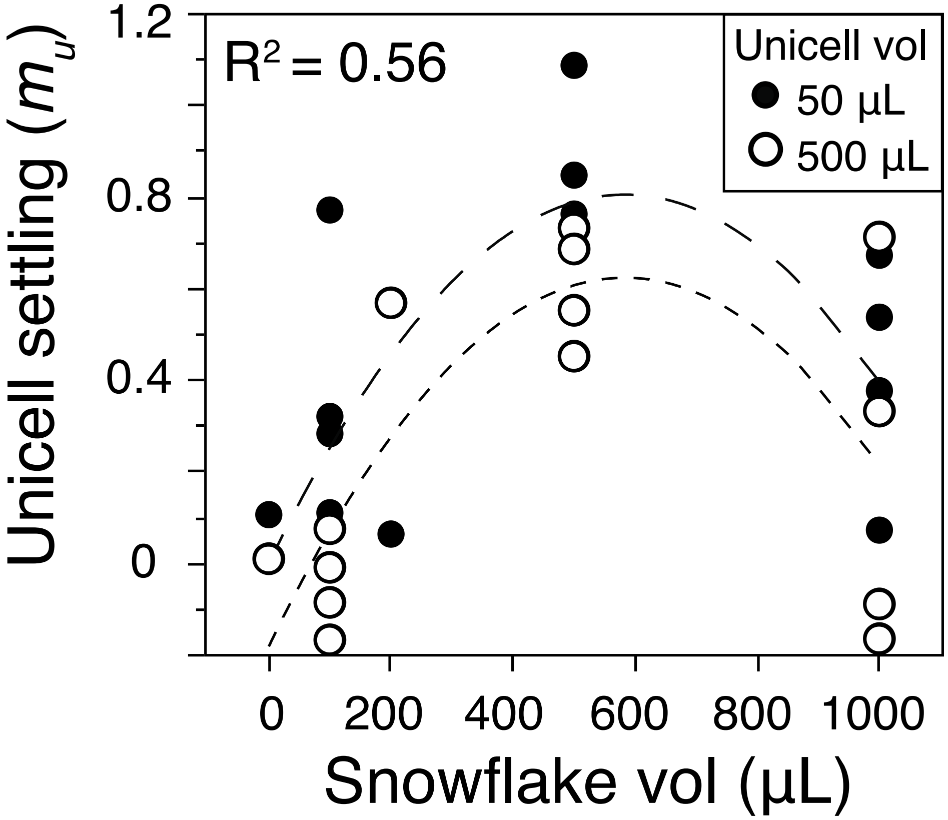

Supplementary Figure 1. Unicell settling depends on snowflake abundance. The benefit to unicells initially increases with snowflake abundance (least-squares regression, linear term: $p < 0.0001$) but declines for high snowflake densities (quadratic term: $p < 0.0001$). In contrast, increased unicell density during settling did not increase unicell settling, but rather marginally reduced unicell settling ($p = 0.051$). Dashed lines show predicted unicell enrichment for low (coarse) and high (fine) unicell additions according to the model $m_U = m_U(0) + V(\mathrm{U}) + V(\mathrm{SF}) + V(\mathrm{SF})^2$, where V is volume, U denotes unicell, SF is snowflake, and $m_U(0)$ is the y-intercept. Model parameters were estimated by ordinary least-squares regression. See Supplementary Data 4 for raw data and complete results of the regression analysis.

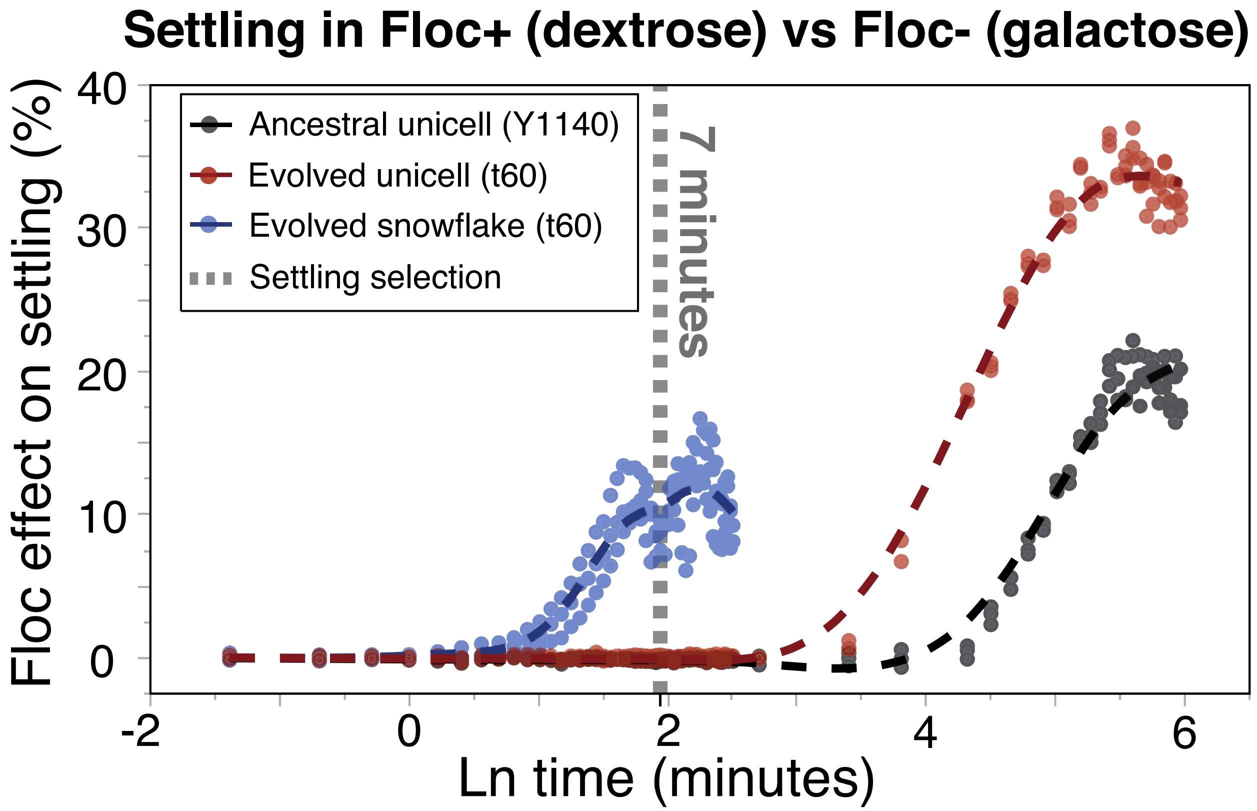

Supplementary Figure 2. Floc positively effects settling in both multicellular and unicellular strains, including the ostensibly non-flocculent ancestor Y-1140. Flocculation accelerates settling over a period relevant to selection (7 minutes) in snowflake cultures (Tukey's HSD: $p < 0.0001$), whereas there is no significant difference between unicell cultures settled under low- or high-flocculation conditions ($p = 0.999$; Supplementary Data 6). However, during extended settling (6.5 hours), evolved unicells settle more rapidly than the ancestor in high-flocculation cultures (Tukey's HSD: $p= 0.0036$), yet there was no significant difference between ancestral and evolved unicells when flocculation was disrupted ($p = 0.51$). Floc effect on settling was quantified as the difference in percent reduction in culture turbidity between high- and low-flocculation conditions. Note that time is in log(minutes) in order to allow visual comparisons of strains with dramatically different settling rates.
